# CXCL1 contributes to IL-6 expression in osteoarthritis and rheumatoid arthritis synovial fibroblasts by CXCR2, c-Raf, MAPK, and AP-1 pathway

**DOI:** 10.1186/s13075-020-02331-8

**Published:** 2020-10-21

**Authors:** Sheng-Mou Hou, Po-Chun Chen, Chieh-Mo Lin, Mei-Ling Fang, Miao-Ching Chi, Ju-Fang Liu

**Affiliations:** 1 Department of Orthopedic Surgery, Shin Kong Wu Ho-Su Memorial Hospital, Taipei, 111 Taiwan; 2 Translational Medicine Center, Shin-Kong Wu Ho-Su Memorial Hospital, Taipei, 111 Taiwan; 3 Department of Biotechnology, College of Medical and Health Science, Asia University, Taichung, 413 Taiwan; 4 Department of Medical Research, China Medical University Hospital, China Medical University, Taichung, 404 Taiwan; 5 Department of Nursing, Chang Gung University of Science and Technology, Puzi City, 613 Chiayi County Taiwan; 6 Division of Pulmonary and Critical Care Medicine, Chang Gung Memorial Hospital, Puzi City, 613 Chiayi County Taiwan; 7 Graduate Institute of Clinical Medical Sciences, College of Medicine, Chang Gung University, Taoyuan, 333 Taiwan; 8 Center for Environmental Toxin and Emerging-Contaminant Research, Cheng Shiu University, Kaohsiung, 833 Taiwan; 9 Super Micro Research and Technology Center, Cheng Shiu University, Kaohsiung, 833 Taiwan; 10 Chronic Disease and Health Promotion Research Center, Chang Gung University of Science and Technology, Puzi City, 613 Chiayi County Taiwan; 11 Division of Pulmonary and Critical Care Medicine, Chiayi Chang Gung Memorial Hospital, Puzi City, 613 Chiayi County Taiwan; 12 Department of Respiratory Care, Chang Gung University of Science and Technology, Puzi City, 613 Chiayi County Taiwan; 13 School of Oral Hygiene, College of Oral Medicine, Taipei Medical University, No. 250 Wu-Hsing Street, Taipei, 110 Taiwan

**Keywords:** CXCL1, Osteoarthritis, Rheumatoid arthritis, IL-6

## Abstract

**Background:**

Osteoarthritis (OA) and rheumatoid arthritis (RA) are common joint disorders that are considered to be different diseases due to their unique molecular mechanisms and pathogenesis. Chemokines and their corresponding receptors have been well characterized in RA progression, but less so in OA pathogenesis.

**Methods:**

The human primary synovial fibroblasts (SFs) were obtained from human OA and RA tissue samples. The Western blot and qPCR were performed to analyze the expression levels of CXCL1, as well as CXCL-promoted IL-6 expression in both OASFs and RASFs. The signal cascades that mediate the CXCL1-promoted IL-6 expression were identified by using chemical inhibitors, siRNAs, and shRNAs.

**Results:**

Here, we found that both diseases feature elevated levels of CXCL1 and interleukin (IL)-6, an important proinflammatory cytokine that participates in OA and RA pathogenesis. In OASFs and RASFs, CXCL1 promoted IL-6 expression in a dose- and time-dependent manner. In OASFs and RASFs overexpressing CXCL1 or transduced with shRNA plasmid, IL-6 expression was markedly upregulated. CXCR2, c-Raf, and MAPKs were found to regulate CXCL1-induced IL-6 expression in OASFs and RASFs. Finally, CXCL1 triggered the transcriptional activities of c-Jun (which regulates the expression of proinflammatory proteins) in OASFs and RASFs.

**Conclusions:**

Our present work suggests that the CXCL1/CXCR2 axis helps to orchestrate inflammatory responses in OA and RA SFs.

## Introduction

Chemokines and chemokine receptors are critical players in the disease processes of two inflammatory joint diseases: rheumatoid arthritis (RA) and osteoarthritis (OA) [1]. Chemokines are abundant in RA synovial fluid, while OA synovial fluid also reveals the presence of chondrocytes, synovial cells, and other cells capable of both expressing and responding to chemokines [2–4]. Both diseases are characterized by the extravasation of leukocytes from the vascular endothelium into the synovial tissue, a process that involves numerous chemokines and their receptors acting as synovial chemotactic mediators [5].

Chemokines are well recognized for their ability to recruit different leukocytes [6] and for their involvement in the migration of circulating cells into or within tissue [7, 8]. Chemokines have been classified by structure and function into four groups: CXC, CC, C, and CX3C [9]. Chemokines can be either homeostatic or inflammatory, or display both qualities simultaneously. Homeostatic chemokines are constitutively produced. Their critical role requires them to maintain physiological traffic and enable homing of cells that largely belong to a specific immune system. In contrast, inflammatory cytokines are produced in response to inflammation in tissue [10].

Chemokine (CXC motif) ligand 1 (CXCL1) acts as a key chemoattractant for neutrophils by binding specifically to its corresponding G-protein-coupled receptor chemokine (CXC motif) receptor 2 (CXCR2) [11, 12]. CXCL1 modulates angiogenesis, tumorigenesis, and wound healing [13]. In general, CXCL1 levels are extremely low under normal physiological conditions and greatly increased during inflammatory conditions. CXCL1 expression appears to be increased in RA and OA patients [14]. Previous research has reported that CXCL1 contributes to the ingress of neutrophils into the RA joint [15] and induce hypertrophy and apoptosis of chondrocytes [16]. According to this evidence, CXCL1 plays a pivotal role in RA and OA pathogenesis.

This study details how we found that CXCL1 promoted interleukin (IL)-6 expression in RA and OA synovial fibroblasts (SFs), worsening the inflammatory status in the joints of both diseases. We have elucidated the molecular mechanisms involved in the increase in IL-6 expression caused by CXCL1 incubation in SFs, which was regulated by its receptor CXCR2, c-Raf, and MAPK signaling components, and activator protein-1 (AP-1) transcriptional activation. Our findings help to explain the pathogenesis of arthritis.

## Materials and methods

### Materials

Dulbecco’s modified Eagle’s medium (DMEM), fetal bovine serum (FBS), penicillin-streptomycin solution, 2 mM l-glutamine, lipofectamine 2000, and TRIzol were purchased from Invitrogen (Carlsbad, CA, USA). Cell culture dishes, 6-well, and 12-well plates were purchased from Greiner Bio-One (Frickenhausen, Germany). Polyvinyldifluoride (PVDF) membrane and an Immobilon Western Chemiluminescent HRP Substrate detection system were purchased from Millipore (Billerica, MA, USA). All of the primary antibodies specific for IL-6 (sc-28343), CXCR2 (sc-32780), c-Raf (sc-7267), MEK (sc-6250), ERK (sc-514302), JNK (sc-7345), p38 (sc-81621), c-Jun (sc-166540), and β-actin (sc-47778) were purchased from Santa Cruz Biotechnology (Santa Cruz, CA, USA). Polyclonal rabbit antibodies specific for phosphorylated forms of c-Raf (#9427), MEK (#3958), ERK (#4376), JNK (#9255), p38 (#9216), and c-Jun (#2361) were purchased from Cell Signaling and Neuroscience (Danvers, MA, USA). All inhibitors against signal pathway components were purchased from Sigma-Aldrich (St. Louis, MO, USA). Recombinant human CXCL1 was obtained from PeproTech (Rocky Hill, NJ, USA). All small interfering RNAs (siRNAs) were purchased from Santa Cruz Biotechnology (Santa Cruz, CA, USA).

### Cell culture

Written informed consent was obtained from all study participants, and the study was approved by the Institutional Review Board of Shin Kong Wu Ho-Su Memorial Hospital (20140712R). Primary SFs were isolated from tissue with OA patients who received knee replacement surgery, using previously described methods [17, 18]. The NSFs and RASFs were purchased from Cell Applications, Inc. (San Diego, CA, USA) and Riken cell bank (Ibaraki, Japan), respectively. Fresh synovial tissues were finely minced and digested in DMEM containing 2 mg/ml type II collagenase (Sigma-Aldrich, St. Louis, MO, USA) for 4 h at 37 °C and under 5% CO_2_. The SFs were maintained in complete DMEM with 10% FBS, penicillin-streptomycin solution, and 2 mM l-glutamine at 37 °C with 5% CO_2_.

### Real-time quantitative polymerase chain reaction (qPCR)

Cells grown in 6-well plates were treated as described in the figure legends, and total RNA was extracted from the cells using TRIzol reagent (Invitrogen) according to the manufacturer’s instructions. The quantity and purity of RNA were assessed using a NanoDrop ND 1000 (Thermo Fisher Scientific, Wilmington, DE, USA). The quality of RNA was further examined by agarose gel electrophoresis. Subsequently, 1 μg of mRNA was subjected to reverse transcription to produce complementary DNA (cDNA).

qPCR was prepared using SYBR Green (KAPA Biosystems, Woburn, MA, USA), according to the manufacturer’s protocol. The primers used in qPCR analysis (human IL-6, CXCL1, and GAPDH) were purchased from Sigma-Aldrich. Reactions were carried out in triplicate using StepOnePlus (Applied Biosystems, Foster City, CA, USA). The cycling conditions were 10 min of polymerase activation at 95 °C followed by 40 cycles at 95 °C for 15 s and at 60 °C for 60 s. The threshold was set above the non-template control background and within the linear phase of target gene amplification, to calculate the cycle number at which the transcript was detected (denoted as CT).

### Western blot analysis

The total cell lysates were extracted from the cells grown in 6-well plates and treated as described in the figure legends. Proteins were resolved using SDS-polyacrylamide gel electrophoresis and transferred to Immobilon PVDF membranes. The blots were blocked with 5% BSA for 1 h at room temperature and then probed using primary antibodies (1:5000 for β-actin; 1:1000 for all of the others) for 1 h at room temperature. After three washes, the blots were incubated with secondary antibodies (1:1000) for 1 h at room temperature. Finally, the blots were photographed with enhanced chemiluminescence using a ChemiDoc-It® Imaging System (UVP Inc., Upland, CA, USA). Quantitative data were obtained using ImageJ software (National Institutes of Health, USA). Each band was separately selected and with the ROI tool and “Gels” function, followed by quantification of the peak area of obtained histograms. The densitometric data of IL-6 protein was normalized to β-actin and the other phosphorylated proteins were normalized to corresponding total proteins, respectively.

### Immunofluorescence staining

Cells grown in chamber slides were subjected to immunofluorescence staining. In brief, the treated cells were fixed with 4% paraformaldehyde at room temperature. Thirty minutes later, 5% nonfat milk in phosphate buffer saline (PBS) containing 0.25% Triton X-100 was added to the cells. The cells were then incubated in rabbit anti-c-Jun (1 : 100) and fluorescein isothiocyanate-(FITC)-conjugated goat anti-rabbit secondary antibodies (1:500; Leinco Technology Inc., St. Louis, MO, USA) for 1 h. FITC was detected using a Zeiss fluorescence microscope.

### Statistics

All values are reported as the mean ± standard error of the mean (SEM). A statistical comparison between two samples was performed using the Student’s *t* test. Statistical comparisons of more than two groups were performed using one-way analysis of variance (ANOVA) followed by Fisher’s least significant difference (LSD) post hoc test. In all comparisons, *p* < 0.05 was considered significant.

## Results

### CXCL1 contributes to IL-6 expression in both OA and RA SFs

**Fig. 1:**
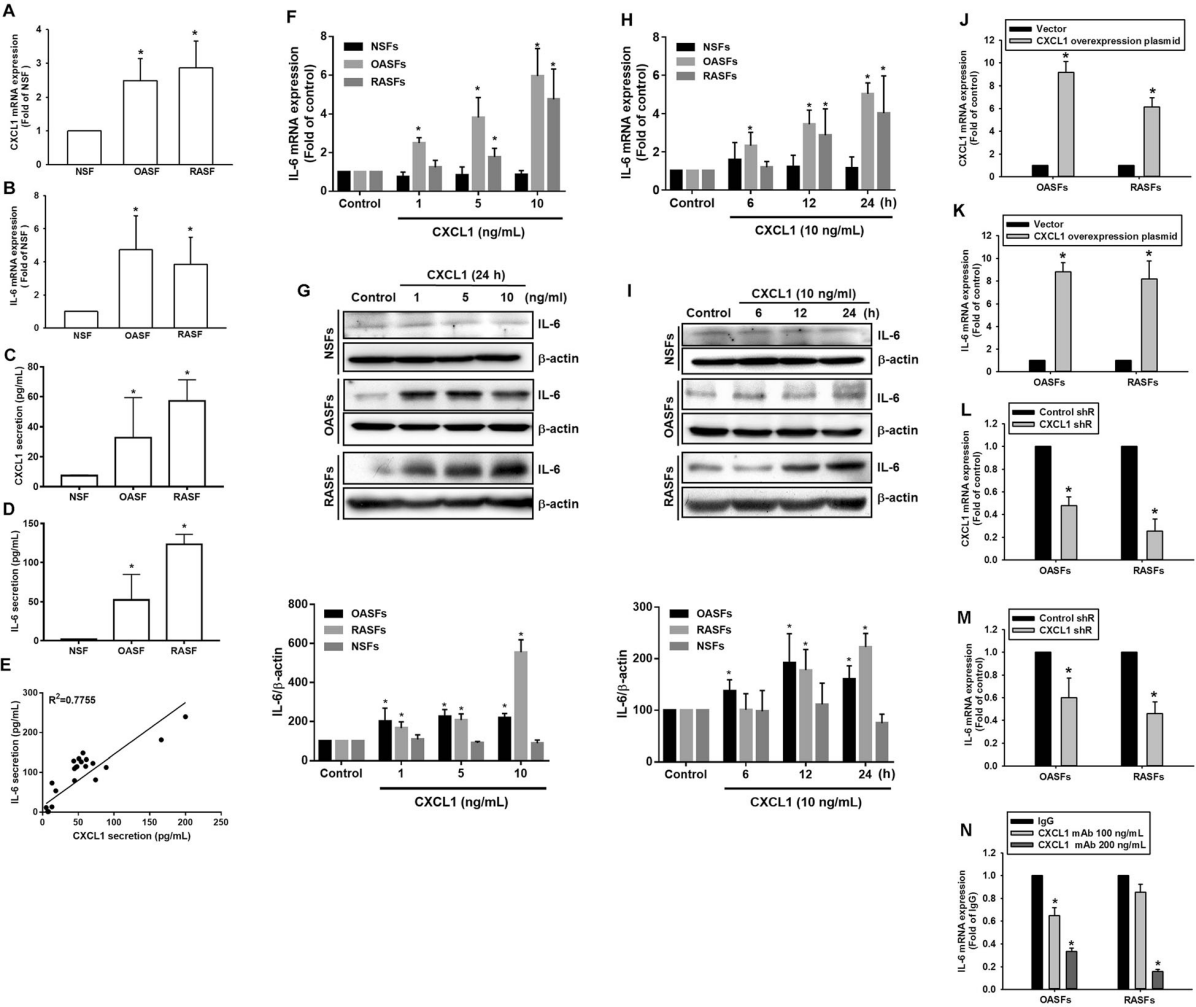
Elevated CXCL1 expression contributes to IL-6 expression in OASFs and RASFs. **a**, **b** The expression levels of CXCL1 and IL-6 were investigated using the qPCR assay. **c**–**e** The protein secretion levels of CXCL1 (**c**) and IL-6 (**d**) were analyzed by using ELISA assay. The correlation between CXCL1 and IL-6 secretion levels from the same specimens was shown in **e**. **f**, **g** SFs isolated from all study participants were incubated with various concentrations (0, 1, 5, and 10 ng/mL) of CXCL1 for 24 h. Total RNA and cell lysates were extracted from SFs and assessed for IL-6 expression using qPCR and Western blot assays. The quantification of Western blot is provided in the lower panel. **h**, **i** OASFs and RASFs were treated with CXCL1 (10 ng/mL) for different time intervals (control, 6, 12, and 24 h), and IL-6 expression levels were determined by qPCR and Western blot. The quantification of Western blot is provided in the lower panel. **j**–**m** OASFs and RASFs were transduced with CXCL1 overexpressing or shRNA plasmids for 24 h, then CXCL1 and IL-6 expression were determined by qPCR. **k** OASFs and RASFs were treated with CXCL1 neutralized antibody (100 and 200 ng/mL) for 24 h, and IL-6 expression levels were analyzed by qPCR. (In the above experiments, NSFs; *n* = 8, OASFs; *n* = 10, RASFs; *n* = 10). Results are expressed as the mean ± SEM. In experiments involving more than two groups, statistical analysis was conducted by using one-way ANOVA followed by Fisher’s LSD post hoc comparisons tests. *p < 0.05 compared with the NSF group (**a**–**d**), control group (**f**–**i**). In experiments involving two groups, *p < 0.05 compared with vector group (**j**, **k**), control shR group (**l**, **m**), or IgG group (**n**)

Previous research has reported an increase in CXCL1 in chondrocytes collected from OA and RA specimens [14]. We quantified levels of CXCL1 expression in SFs from OA and RA patients, as well as normal synovial fibroblasts (NSFs) from healthy controls and found higher CXCL1 expression in both OASFs and RASFs compared with NSFs (Fig. 1a). IL-6, a multifunctional cytokine with a critical role in the pathogenesis of RA [19], has also been implicated in inflammatory response and cartilage loss in OA patients [20]. Levels of IL-6 expression were also higher in OASFs and RASFs than in NSFs, suggesting an association between CXCL1 and IL-6 in OA and RA pathogenesis (Fig. 1b). The protein secretions of CXCL1 and IL-6 in these SFs were also evaluated by ELISA (Fig. 1c, d), and obvious correlation between secretion levels of CXCL1 and IL-6 was found (Fig. 1e). We then investigated whether CXCL1 regulates IL-6 expression in NSFs, OASFs, and RASFs. IL-6 expression was dramatically increased in response to CXCL1 incubation in a dose- and time-dependent manner in OASFs and RASFs but not NSFs, as assessed by qPCR and Western blot analyses (Fig. 1f–i). To confirm the role of CXCL1 in the regulation of IL-6 expression, OASFs and RASFs transduced with overexpressing CXCL1 and shRNA plasmids were subjected to evaluate IL-6 expression. CXCL1 overexpression markedly promoted IL-6 expression, while knockdown of CXCL1 inhibited IL-6 expression in SFs established from OA and RA patients (Fig. 1j–m). Finally, pretreatment with CXCL1 antibodies dramatically abolished IL-6 expression in OASFs and RASFs (Fig. 1n). The evidence demonstrates that CXCL1 contributes to OA and RA pathogenesis, which is mediated by IL-6 expression in SFs.

### CXCR2 is responsible for IL-6 expression after CXCL1 treatment in OASFs and RASFs

**Fig. 2:**
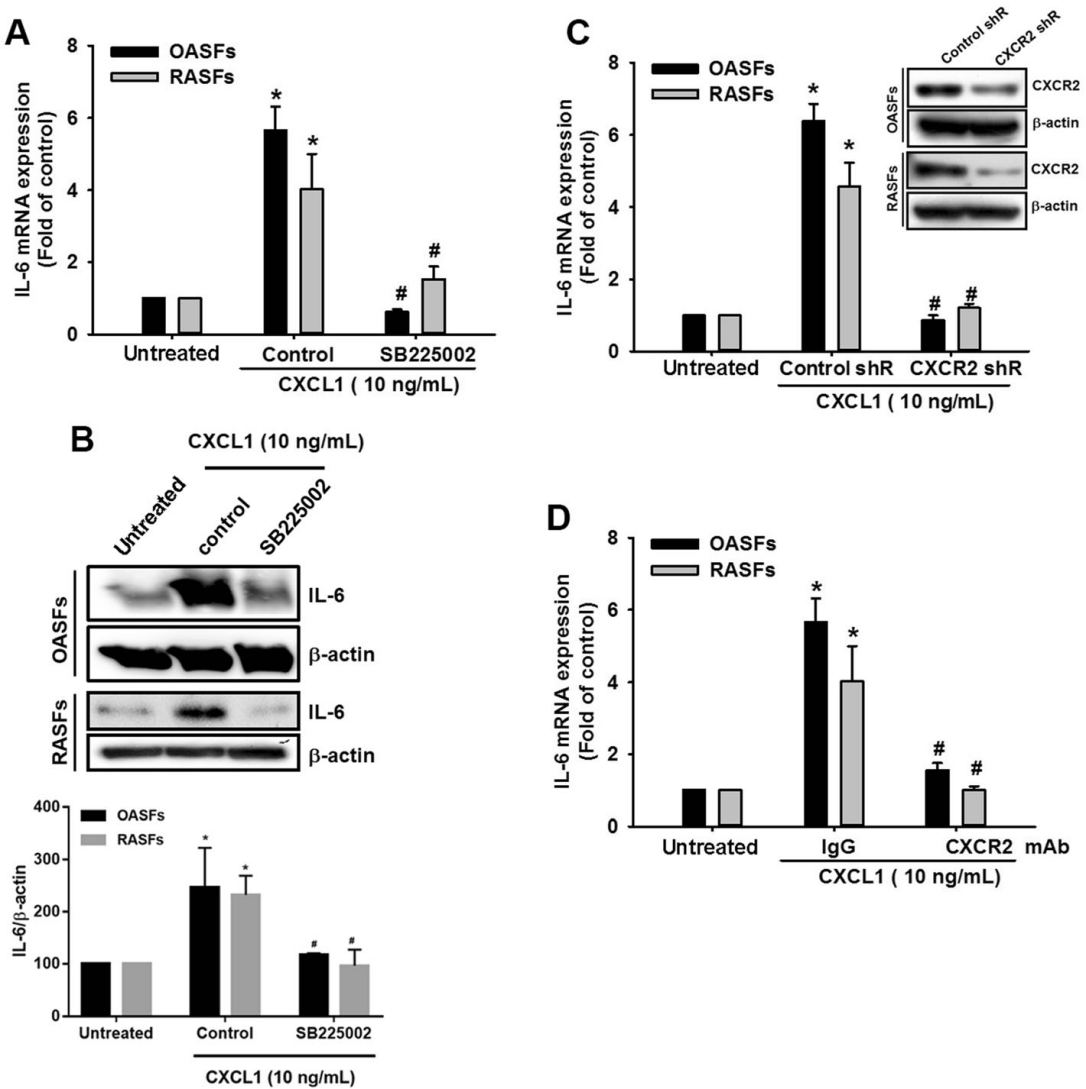
CXCR2 is responsible for CXCL1-increased IL-6 expression in OASFs and RASFs. **a**, **b** OASFs and RASFs were pretreated with a CXCR2 inhibitor (SB225002, 5 μM) for 1 h, then incubated with CXCL1 (10 ng/mL) for 24 h. IL-6 expression levels were quantified by qPCR and Western blot. The quantification of Western blot is provided in the lower panel. **c** OASFs and RASFs were transfected with CXCR2 shRNA plasmids for 24 h (the knockdown efficiency was presented by Western blot), then treated with CXCL1 for 24 h. IL-6 expression levels were determined by qPCR. **d** The OASFs and RASFs were incubated with CXCR2 antibody, followed by treated with CXCL1 for 24 h. IL-6 expression levels were determined by qPCR. (In the above experiments, OASFs; *n* = 10, RASFs; *n* = 10). Results are expressed as the mean ± SEM. Statistical analysis was conducted by using one-way ANOVA followed by Fisher’s LSD post hoc comparisons tests. *p < 0.05 compared with the respective groups in all figures (untreated); ^#^*p* < 0.05 compared to the groups with control (**a**, **b**), control shR (**c**), and IgG (**d**) pretreatment followed by CXCL1 incubation

In regard to the need for CXCR2 for CXCL1 to perform its effect on target cells, we examined whether CXCR2 is required for IL-6 expression in response to CXCL1 treatment. In the presence of SB225002, a CXCR2 inhibitor, CXCL1-induced IL-6 expression was clearly inhibited in both OASFs and RASFs (Fig. 2a, b). Using CXCR2 shRNA or neutralized antibody to block the CXCL1/CXCR2 axis also reversed CXCL1-induced IL-6 expression in both OASFs and RASFs (Fig. 2c, d), suggesting that the CXCL1/CXCR2 axis is responsible for increased levels of IL-6 expression in OASFs and RASFs.

### Activation of c-Raf mediates IL-6 expression in response to CXCL1 treatment in OASFs and RASFs

**Fig. 3:**
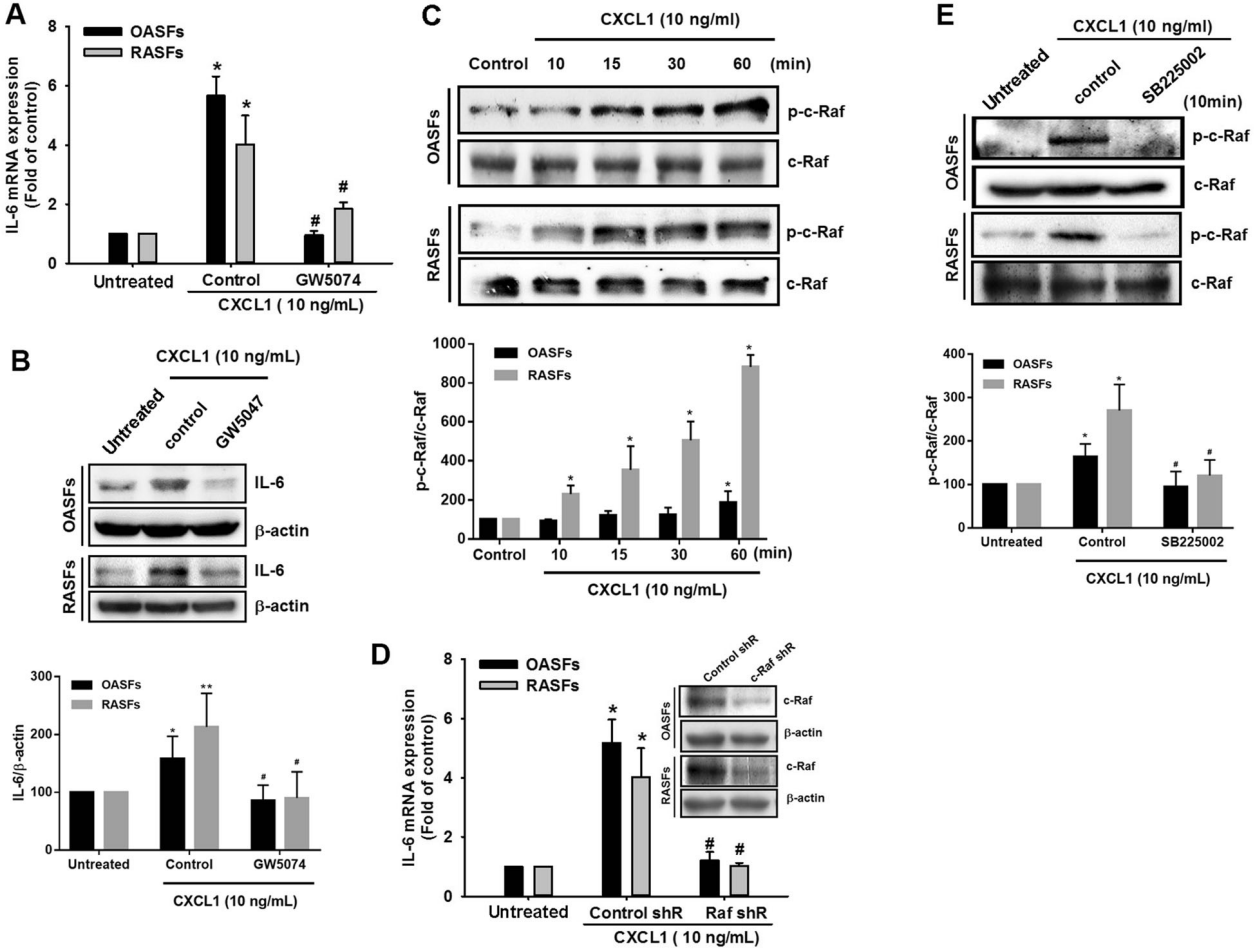
c-Raf is involved in CXCL1-increased IL-6 expression in OASFs and RASFs. **a**, **b** OASFs and RASFs were pretreated with a c-Raf inhibitor (GW5047, 5 μM) for 1 h, then incubated with CXCL1 (10 ng/mL) for 24 h. IL-6 expression levels were quantified by qPCR and Western blot. The quantification of Western blot is provided in the lower panel. **c** OASFs and RASFs were incubated for different periods of time (control, 10, 15, 30, and 60 min). The cell lysates were collected, and phosphorylated c-Raf was determined by Western blot. The quantification of Western blot is provided in the lower panel. **d** OASFs and RASFs transfected with c-Raf shRNA were further incubated with CXCL1 (10 ng/mL) for 24 h, then IL-6 expression was examined by qPCR. (The knockdown efficiency was presented by Western blot). **e** OASFs and RASFs were pretreated with a CXCR2 inhibitor (SB225002, 5 μM) and examined for phosphorylation of c-Raf in the presence of CXCL1. The quantification of Western blot is provided in the lower panel. (In the above experiments, OASFs; *n* = 10, RASFs; *n* = 10). Results are expressed as the mean ± SEM. Statistical analysis was conducted by using one-way ANOVA followed by Fisher’s LSD post hoc comparisons tests. *p < 0.05 compared with the respective groups in all figures (control and untreated); ^#^*p* < 0.05 compared to the groups with control (**a**, **b**, **e**) and control shR (**d**) pretreatment followed by CXCL1 incubation

c-Raf is a typical signal transducer involved in chemokine receptors activation such as CXCR2 [21–23]. Pretreatment of cells with a c-Raf inhibitor (GW5074) significantly reversed IL-6 expression in the presence of CXCL1 in OASFs and RASFs (Fig. 3a, b). We found that CXCL1 treatment increased c-Raf phosphorylation in OASFs and RASFs (Fig. 3c). The involvement of c-Raf in CXCL1-promoted IL-6 expression was confirmed through transfection with c-Raf shRNA (Fig. 3d). When we checked whether c-Raf activation is a downstream effector of the CXCL1/CXCR2 axis, we found that pretreatment with the CXCR2 inhibitor attenuated c-Raf phosphorylation in both OASFs and RASFs (Fig. 3e). This evidence conclusively indicates that c-Raf activation is responsible for CXCL1-promoted IL-6 expression in OASFs and RASFs.

### Activation of MAPK signaling participates in CXCL1-induced promotion of IL-6 expression in OASFs and RASFs

**Fig. 4:**
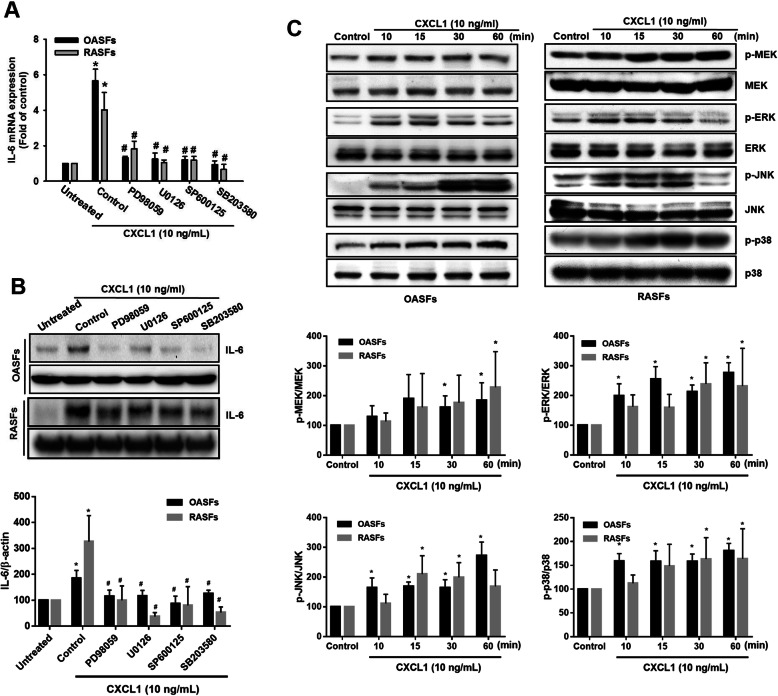
MAPK activation is required for IL-6 expression in response to CXCL1 in OASFs and RASFs. **a**, **b** OASFs and RASFs were pretreated with different inhibitors that block activation of various MAPK signal components (ERK, PD98059, 5 μM; MEK, U0126, 3 μM; JNK, SP600125, 3 μM; p38, SB203580, 5 μM) for 1 h, then incubated with CXCL1 (10 ng/mL) for 24 h. IL-6 expression was examined by qPCR and Western blot. The quantification of Western blot is provided in the lower panel. **c** The cell lysates were collected from OASFs and RASFs as described in Fig. 3c, and Western blot analysis assessed MEK, ERK, JNK, and p38 activation by monitoring the phosphorylated forms of these proteins. The quantification of Western blot is provided in the lower panel. (In the above experiments, OASFs; *n* = 10, RASFs; *n* = 10). Results are expressed as the mean ± SEM. Statistical analysis was conducted by using one-way ANOVA followed by Fisher’s LSD post hoc comparisons tests. *p < 0.05 compared with the respective groups in all figures (control and untreated); ^#^*p* < 0.05 compared to the groups with control pretreatment followed by CXCL1 incubation

The MAPK pathway comprises several key signaling components, including ERK, JNK, and p38, which activate c-Raf signaling and impact cancer progression [24]. We therefore examined whether MAPKs are required for CXCL1 effects in OASFs and RASFs. Our results indicated that pretreatment with MAPK inhibitors (PD98059, ERK; U0126, MEK; SP600125, JNK; SB203580, p38) abolishes CXCL1-induced promotion of IL-6 expression in OASFs and RASFs (Fig. 4a, b). We also identified MAPK signal activation by detecting phosphorylation of MAPK components, and treatment with CXCL1 induced phosphorylated MEK, ERK, JNK, and p38 proteins in OASFs and RASFs (Fig. 4c). These data prove that MAPK activation is involved in CXCL1-promoted IL-6 expression in OASFs and RASFs.

### The AP-1 transcription factor mediates CXCL1-induced promotion of IL-6 expression in OASFs and RASFs

**Fig. 5:**
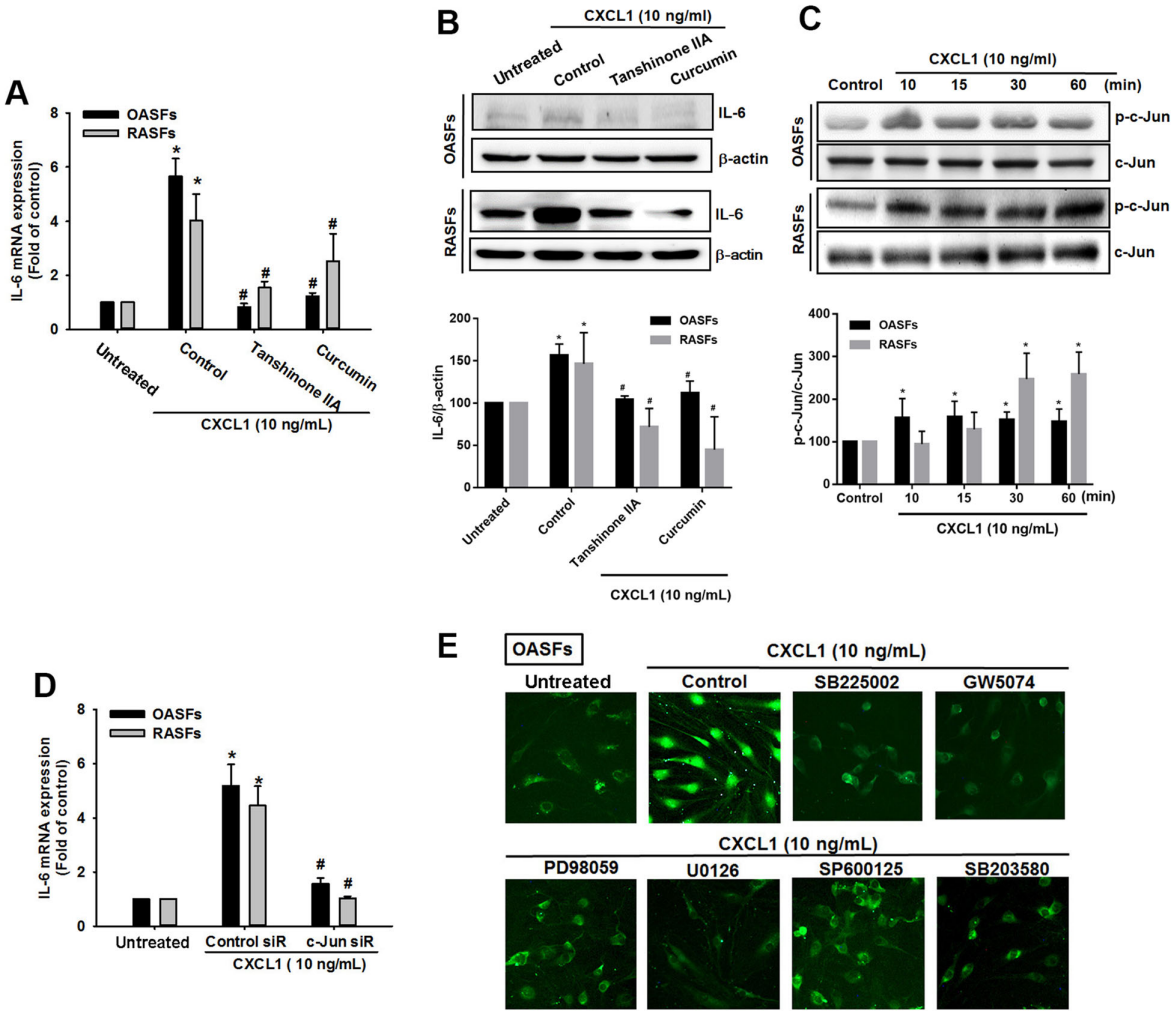
AP-1 transcription factor mediates IL-6 expression in response to CXCL1 in OASFs and RASFs. **a**, **b** OASFs and RASFs were pretreated with different inhibitors against AP-1 activation (tanshinone, 5 μM; curcumin, 3 μM) for 1 h, then incubated with CXCL1 (10 ng/mL). IL-6 expression was examined by qPCR and Western blot. The quantification of Western blot is provided in the lower panel. **c** The cell lysates were collected from OASFs and RASFs as described in Fig. 3c, then c-Jun phosphorylation was investigated by Western blot. The quantification of Western blot is provided in the lower panel. **d** OASFs and RASFs were transfected with c-Jun siRNA for 24 h, then treated with CXCL1 (10 ng/mL) for 24 h. IL-6 expression was analyzed by qPCR. **e** OASFs were pretreated with inhibitors that target CXCR2 (SB225002, 5 μM), c-Raf (GW5047, 5 μM), ERK (PD98059, 5 μM), MEK (U0126, 3 μM), JNK (SP600125, 3 μM), and p38 (SB203580, 5 μM) for 1 h, then incubated with CXCL1 (10 ng/mL) for a further 24 h. Immunofluorescence staining using the c-Jun antibody monitored nuclear translocation. (In the above experiments, OASFs; *n* = 10, RASFs; *n* = 10). Results are expressed as the mean ± SEM. Statistical analysis was conducted by using one-way ANOVA followed by Fisher’s LSD post hoc comparisons tests. *p < 0.05 compared with the respective groups in all figures (control and untreated); ^#^*p* < 0.05 compared to the groups with control (**a**) and control siR (**b**) pretreatment followed by CXCL1 incubation

AP-1 plays a critical role in inflammatory responses; AP-1 activation is involved in OA and RA pathogenesis [25, 26], and AP-1 transcriptional activation stimulates IL-6 expression in RASFs [27]. We investigated the role of AP-1 in IL-6 expression after CXCL1 treatment in OASFs and RASFs. Pretreatment with AP-1 inhibitors (tanshinone IIA and curcumin) blocked IL-6 expression in response to CXCL1 treatment in OASFs and RASFs (Fig. 5a, b). When AP-1 activation was examined by phosphorylation of c-Jun, the results revealed that CXCL1 promoted c-Jun phosphorylation in OASFs and RASFs (Fig. 5c). When we used c-Jun siRNA to confirm our hypothesis, the evidence indicated that transfection with c-Jun siRNA inhibited CXCL1-induced promotion of IL-6 expression in OASFs and RASFs (Fig. 5d). Finally, we examined AP-1 activation by monitoring its nucleus translocation. As shown in Fig. 5e, the nucleus translocation of AP-1 was increased in the CXCL1-treated cells; however, pretreatment with pathway inhibitors involved in CXCL1 effects dramatically diminished this phenomenon, confirming involvement of the CXCR2, c-Raf, MAPK, and AP-1 signaling transduction pathways in response to CXCL1 treatment.

## Discussion

Abundant evidence has implicated chemokines in pathogenesis and improved them as therapeutic targets of RA [28] as well as OA [29]. RA is an autoimmune disease, in which infiltration of inflammatory cells into the joint leads to chronic inflammation and articular destruction [30], while OA is considered to be a degenerative disease of the cartilage with a low-grade inflammatory status [31]. In this study, we demonstrate how upregulation of the CXCL1/CXCR2 axis in both OASFs and RASFs contributes to inflammatory cytokine IL-6 expression, revealing the pivotal role of CXCL1/CXCR2 activation in chronic inflammation of synovial fibroblasts. Although these two common joint diseases are characterized by different mechanisms of pathogenesis and molecular conception, SFs are a common compartment of inflammation and joint erosion in these diseases [32]. Furthermore, a previous report indicated common pathogenic mechanisms shared by SFs in both diseases [33]. Our present finding could discover new therapeutic targets in inflammatory joint disease treatment.

Previous research has identified upregulated CXCL1 expression in chondrocytes isolated from OA and RA specimens [14]. In our present study, a high level of CXCL1 expression was detected in both OASFs and RASFs. A previous study showed that the fundamental effect of the CXCL1/CXCR2 axis on the recruitment of neutrophils means that it exerts an important role in RA progression [34]. Furthermore, this report found a higher expression level of CXCL1 in RA synovial fluid compared with OA synovial fluid. Our current work focuses on the molecular mechanism involved in RA and OA pathogenesis in cell culture model of synovial fibroblasts. In accordance with previous report, we found secretion level of CXCL1 was higher in culture media of RASFs than OASFs and NSFs. The CXCL1/CXCR2 axis is also recognized as a therapeutic target, as it plays a critical role in the activation of neutrophils involved in inflammatory diseases including RA [35]. For instance, the CXCR2/CXCR1 antagonist SCH563705 greatly attenuated disease severity in a mouse model of RA [36]. Another CXCR2 inhibitor, DF2162, significantly attenuated adjuvant-induced polyarthritis in rats [37]. Conversely, CXCL1/CXCR2 signaling is also associated with chondrocyte phenotypic stability [38], and upregulation of CXCL1 can influence the differentiation of articular chondrocytes, leading to articular chondrocyte hypertrophy [39], revealing the critical role of CXCL1 during the progression of OA. Several clinical trials have provided evidence implicating the CXCL1/CXCR2 axis as a therapeutic target for asthma, bronchiectasis, and cancer, with promising effects in chronic obstructive pulmonary disorder (COPD) [40]. The clinical investigation of CXCL1/CXCR2 agonists in RA or OA deserves to be explored.

IL-6 plays a critical role when immune cells encounter bacteria or other microorganisms [41, 42]. Dysregulation of IL-6 leads to many autoimmune and inflammatory diseases, including RA [43]. Targeting IL-6 with the anti-IL-6 receptor (IL-6R) antibody tocilizumab is effective for treating RA [44]. A previous review has confirmed the important role of IL-6 in OA progression [45], which is supported by the finding that IL-6 expression is elevated in OA human synovial fluid and sera [46]. In this report, we found that CXCL1/CXCR2 activation promoted IL-6 expression in a time-dependent manner, suggesting IL-6 is immediate-early gene in OA and RA pathogenesis [47]. Furthermore, CXCL1 dose-dependently induced IL-6 expression in OASFs and RASFs with most induction at 10 ng/mL CXCL1 treatment, while treatment with 50 ng/mL CXCL1 showed minor effect of IL-6 expression (data not shown), suggesting bidirectional effects of CXCL1 in the context of CXCL1 concentration. Finding that CXCL1 and CXCR2 have active roles in OA and RA pathogenesis via inflammatory SFs was not surprising, since CXCL1 expression was increased in SFs isolated from OA and RA specimens and CXCL1 promoted IL-6 expression in OASFs and RASFs. Preclinical and clinical investigations are warranted to explore the role of CXCL1/CXCR2 in OA and RA progression.

CXCR2, a G-protein-coupled chemokine receptor, has been identified as a specific receptor for CXCL1 to exert its biological functions [48–50]. CXCR2 activates multiple signaling pathways, such as the PI3K/Akt, PLC/PKC, MAPK, and JAK/STAT3 pathways [40]. CXCR2 expression activates MAPKs ERK and p38, but not JNK [51]. MAPK proteins are a family of serine/threonine protein kinases commonly activated by proinflammatory cytokine stimulation, which in turn regulate the production of inflammation mediators [52]. Previous study discusses the cross-talk between IL-1 and IL-6 in RASFs, by which enhancing proinflammatory signal pathway through p38 MAPK [53]. Our results indicate that the CXCL1/CXCR2 axis triggers activation of MAPK signaling proteins, including MEK/ERK, p38, and JNK. In accordance with previous evidences, our data suggest that MAPK signaling transduction pathways may serve a pivotal role in the amplification of the inflammatory response caused by the CXCL1/CXCR2 axis. Since the Raf–MAPK cascade exerts its regulatory function downstream of cell surface receptors and dysregulated in cancer and other human pathologic disorders [54, 55], revealing this canonical signaling network could develop as pharmacologic inhibitors to block Raf–MAPK signaling for the treatment of chronic joint disease. Furthermore, we found increased phosphorylation of c-Jun following CXCL1 treatment; c-Jun is a major component of the AP-1 transcription factor. Other researchers have reported finding that c-Fos, another major component of the AP-1 transcription factor, was induced in response to CXCL1 injections in spinal cord neurons [56]. It would be useful to detail the mechanisms involved in transcriptional activation triggered by CXCL1/CXCR2.

## Conclusions

In summary, we found signaling pathways involved in CXCL1-induced promotion of IL-6 in human OASFs and RASFs, which was mediated through CXCR2, c-Raf, MAPK, and AP-1. Fully understanding the signaling pathways will improve our knowledge about the role played by CXCL1 in the progression of OA and RA disease and assist with the discovery of novel therapeutic agents.

## Data Availability

The data sets used and analyzed during the current study are available from the corresponding author on reasonable request.
